# Cognitive Archeology and the Attentional System: An Evolutionary Mismatch for the Genus *Homo*

**DOI:** 10.3390/jintelligence11090183

**Published:** 2023-09-12

**Authors:** Emiliano Bruner

**Affiliations:** Centro Nacional de Investigación sobre la Evolución Humana, Paseo Sierra de Atapuerca 3, 09002 Burgos, Spain; emiliano.bruner@cenieh.es

**Keywords:** human evolution, cognitive evolution, human genus, parietal cortex, attention, stress, anxiety

## Abstract

Brain evolution is a key topic in evolutionary anthropology. Unfortunately, in this sense the fossil record can usually support limited anatomical and behavioral inferences. Nonetheless, information from fossil species is, in any case, particularly valuable, because it represents the only direct proof of cerebral and behavioral changes throughout the human phylogeny. Recently, archeology and psychology have been integrated in the field of cognitive archeology, which aims to interpret current cognitive models according to the evidence we have on extinct human species. In this article, such evidence is reviewed in order to consider whether and to what extent the archeological record can supply information regarding changes of the attentional system in different taxa of the human genus. In particular, behavioral correlates associated with the fronto-parietal system and working memory are employed to consider recent changes in our species, *Homo sapiens*, and a mismatch between attentional and visuospatial ability is hypothesized. These two functional systems support present-moment awareness and mind-wandering, respectively, and their evolutionary unbalance can explain a structural sensitivity to psychological distress in our species.

## 1. Attention and Human Evolution

The origin of the human genus (i.e., the genus *Homo*) is roughly estimated at 2 million years ago, in Africa (Antón and Snodgrass 2012; Antón et al. 2014). Despite the importance we place on such a phylogenetic event, the paucity and incompleteness of the fossil record has hampered, to date, the development of consistent hypotheses on the species—and changes—involved (Wood 2014). In fact, few species do fossilize (scarce phylogenetic information), and those that did can only supply data on their skeletal elements (scarce biological information), with difficulties in species recognition (scarce taxonomic information) and few specimens available (scarce statistical information) (Bruner 2013). Indeed, this situation requires, at least, caution, when firm statements are provided. If the general evolutionary scenario of the human phylogenetic origin is still unclear, the information available on the associated brain changes are, as would be expected, even less conclusive. The field that investigates brain anatomy in fossils is called *paleoneurology*, and deals with the analysis of brain morphology as evidenced by the form and features of the cranial cavity, aided by the use of endocranial molds called *endocasts* (Bruner 2015, 2019). In the second half of the past century, there was a general agreement on the fact that the origin of the human genus was associated with a certain expansion of the fronto-parietal cortex (particularly of the language areas), and with a relative increase in the overall brain size (Tobias 1987). However, after several decades of additional research, we can now state that the situation is, frankly speaking, not so clear (Bruner and Beaudet 2023). The mean increase in brain size is probably a fact, but the fossil record is too scattered to make firm inferences on single specimens, mostly when considering that species, in paleontology, are difficult to establish with sufficient reliability. The situation is similar for what concerns the fronto-parietal expansion: individual variations are consistent, sulcal patterns are difficult to assess from an endocast, and the small samples do not support robust statistical tests. A large part of the neurocranial variability in early humans and archaic hominids is indeed due to size differences only, with scarce evidence of species-specific changes in cortical features or proportions (Bruner et al. 2023a).

However, fossils are not the only informative elements of human history. Archeological evidence deals with the ecological, technological, and social aspects of our evolution, and can supply additional cognitive cues on behavioral aspects of extinct hominids. The term *cognitive archeology* is used to define a field in which cognitive sciences and archeology are integrated in order to provide cognitive models in evolutionary anthropology, mostly by interpreting behavioral evidence associated with extinct populations with theories and hypotheses from current psychology (Wynn and Coolidge 2016). In this case, we cannot analyze the cognitive functions directly, so we must use inferred behaviors as a bridge between the archeological remains and underlying cognitive factors. For example, we can consider the hierarchical models commonly based in broad, narrow and specific cognitive abilities (like the Cattell–Horn–Carroll theory), and investigate whether behavioral data can reveal specific changes in the cognitive structure of extinct human species (Bruner 2022a; Bruner et al. 2023b). This approach, now employed in evolutionary anthropology and prehistory, could actually also be used when dealing with modern (i.e., historical) populations in order to evaluate the effects of distinct cultural environments on the cognitive architecture.

Even when dealing with behavior, however, the key features of human origins are not patent at all. Technology is, in this sense, a central issue, as tool-use and tool-making have always been interpreted as a special human trait (Ambrose 2001). Of course, other animals have some skills in handling and manipulation (e.g., Whiten et al. 1999; Shumaker et al. 2011), but at a level of complexity that is not comparable with the outstanding technological capacity in humans. Actually, I have previously proposed that a “tool” should be intended as an obligatory technological element (compulsory in terms of ecological and cognitive niche), part of a technological network, and integrated in the brain–body schemes (Bruner and Gleeson 2019). In this sense, probably only humans are real “tool-users”, while other animals must be instead interpreted as “object-users” (Bruner 2021). Anyway, there is no doubt that human technology, in its amazing degree of complexity, represents an outstanding specialization, and that is why archeology has been, since ever, interested in the evolution of the cognitive abilities associated with tools. Nonetheless, the increasing archeological record has, once more, generated more disagreements than certainties, when dealing with the phylogenetic origin of such exceptional technological capacity. On the one hand, there is the possibility that other hominids, not belonging to the human genus, could have used stone tools, even if only at a basic technological level. At the same time, it is not clear whether tool-use in early humans may have required particular cognitive skills, beyond the ones described in other living primates. However, after those early and blurred origins around 2 million years ago, there is no doubt that technology became, afterward, a unique key adaptation of our genus. We can probably say that, before 1.7 million years, humans were *occasional* tool-users, then becoming *habitual* tool-users and, finally, after 300,000 years, *obligatory* tool-users (Shea 2017). “Obligatory” means that our ecology and cognition is strictly dependent on extra-somatic peripheral devices (“tools”), which are necessary to scaffold and maintain our mental and economical standards.

**Figure 1:**
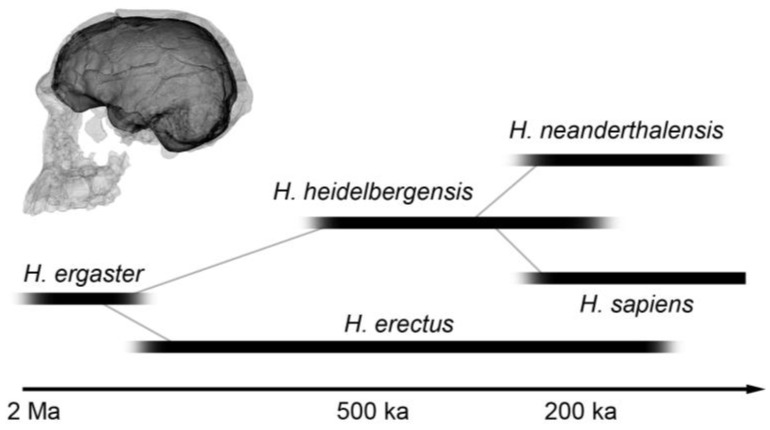
Approximate chronological extension of the human species with consistent paleontological and archeological record (Ma: million years; ka: thousands years). The digital replica shows the skull and endocast of KNM-ER 1813, a specimen assigned to the controversial species *H. habilis*. Redrawn after Bruner and Beaudet (2023).

Following their phylogenetic origin in Africa, humans underwent an evolutionary radiation through three continents, with a diversification that included an uncertain number of species, depending upon the different interpretation of several taxonomic concepts and of the osteological remains. Indeed, the number of human species for which we have modest but fruitful paleontological information are few, say four or five (Figure 1), accompanied by a set of additional and uncertain taxa that are less documented, associated with single excavation sites, or scarcely defined in terms of taxonomy.

Regardless of the precise patterns, timing and composition of our own natural history, we can be reasonably confident that cognitive complexity has been a key factor in our evolutionary adaptations. There are still disagreements on whether our cognitive abilities are unique to our species (in this case, modern humans would have evolved distinctive mental skills that are absent in other species), or are the result of a disproportionate expansion of abilities that are also present in other primates (in this case, the cognitive differences in *Homo sapiens* would be a matter of degree). However, there is no denying that cognitive complexity is definitely extraordinary in our species, and hence we should investigate when and how such complexity evolved.

Among the many cognitive abilities involved in two million years of human evolution, attention has probably played an exceptional role, as a bottleneck for any other mental skills (Rueda 2018; Rueda et al. 2023). Both in terms of evolutionary and psychological networks, attention has been in fact defined as a *limiting factor*, namely, a feature that constrains the execution and evolution of all the other cognitive aspects (Bruner and Colom 2022). Attention is the capacity to sustain a specific cognitive effort in time and space regardless of internal or external competing stimuli, and as such, it determines the actual efficiency of any (e.g., mnemonic, linguistic, visuospatial, analytic, etc.) mental process. Because of its crucial position within the cognitive network, we must suppose that attentional abilities have undergone important changes throughout the evolution of the genus *Homo*.

## 2. Cognitive Archeology and Attention

Current theories on attention suggest that this label could actually represent a heterogeneous system of abilities and neural networks that, albeit integrated and overlapping, rely on distinct circuits and factors. The fronto-parietal attention network has been proposed to be based on 18 anatomical subregions (Scolari et al. 2015), and it is hence expected that attention is a heterogeneous and multifactorial cognitive ability, difficult to frame into stringent definitions or within specific experimental paradigms (MacLeod et al. 2010; Krauzlis et al. 2014; Hommel et al. 2019). A basic model, which is partially supported anatomically, separates *alerting* (arousal and vigilance), *orienting* (selection of stimuli) and *executive control* (decision-making and volitional management) (Petersen and Posner 2012). This tripartite model is well supported on a general neuroanatomical and functional ground, and is useful to stimulate the debate concerning the existence of distinct attentional subsystems, influenced by different biological variables and environmental conditions (Posner 2023; Posner and Rothbart 2023; Rueda et al. 2023). This is particularly important when we recognize that human evolution, as for the evolution of most taxa, is not a linear and gradual progression, but a bushy network formed by alternative, parallel and independent branches. This leads us to two main phylogenetic conclusions. First, distinct hominid lineages may have evolved different attentional abilities. In general, an old-fashioned view (scientifically abandoned half century ago, but unfortunately still in vogue outside the field) put all hominids on a single line, progressing from most primitive to most evolved species. Such progression is, as far as we know, decidedly unlikely, and the array of extinct human species should be interpreted as an admixture of common ancestors and side branches, generating a fuzzy assortment of primitive and derived traits. In this case, the attentional systems of two human lineages are not necessarily “better” or “worse”, more primitive or more evolved, but might be simply “different”. Different may mean that they are independent options, based on different processes and selected by different environmental necessities. The second aspect deals with the fact that attention, being formed by different subsystems or different cognitive skills, can evolve in a mosaic fashion, and not along a homogeneous sequence. Namely, different human lineages may have evolved different “combinations” of attentional skills. This is of course true also when we consider our own intra-specific variability, but, in the case of evolution, differences may definitely be more pronounced. This scenario makes things even harder to decipher because, apart from the difficulties of working with incomplete fossil remains and partial archeological artifacts, we should also consider that extinct humans might have evolved attentional features that stand outside of our own definition of attention, or in some peculiar combinations that we are not able to assess.

With all these caveats in mind, we can investigate the paleontological and archeological record in search of some signal that can reveal that “something” has changed in the attentional system of a given species. Using the tripartite system as a general reference, we can speculate whether those three functions have experienced specific variations in the evolution of the human genus.

### 2.1. Alerting and Orienting

Arousal is probably based on different components (Robbins 1997), and there is evidence of some anatomical specializations in the ascending reticular activating system in our own species (Edlow et al. 2012). However, because of its generalized behavioral effects, and being a key function for the survival of any living organism, specific inferences on possible evolutionary influences on the genus *Homo* are more difficult to provide. Waiting for further information, we can probably hypothesize that, of the three subsystems, it could have undergone the least specialization in our lineage. Namely, it is hard to evaluate whether or not different human species may have had or required different alerting responses.

**Figure 2:**
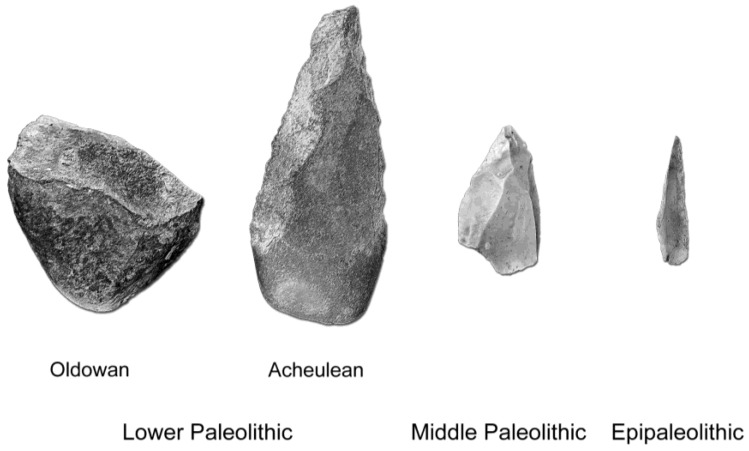
Oldowan choppers, Acheulean handaxes, Mousterian flakes and Epipaleolithic point (not to scale). These tools display a decrease in size and an increase in geometrical and perceptual complexity. In general, however, more complex tools do not substitute the preceding technology, but are added to the general toolkit. Therefore, in later technological stages, there is a general overlap between more archaic and more derived tools. Redrawn after (Bruner et al. 2018c).

Instead, the orienting system may have undergone more targeted changes through human evolution, considering that most human behaviors require a peculiar selection of stimuli. The existence of affordances (sensu Gibson 1979), for example, should suggest that this selection of stimuli was subject to a major rearrangement when “objects” became “tools”, namely, in those species that became habitual tool users, and then even more with those species that turned into obligatory tool users. Affordances are emergent properties associated with mutual relationships between organism and environment, and hence require a specific cognitive scaffold to trigger a given response. Such scaffold largely includes attentional selectivity, following both top-down and bottom-up processes. With this in mind, the first consistent technological change (habitual tool users) is associated with *H. erectus*, or with its African ancestor, *H. ergaster*. In these species, as in the later and more encephalized *H. heidelbergensis*, raw knapped stones (Oldowan choppers; Wynn et al. 2011) were largely integrated into stone tools that were knapped all along the surface, designed intentionally for diverse functions, and with a roughly symmetric morphology (Acheulean handaxes; Wynn and Gowlett 2018) (Figure 2).

**Figure 3:**
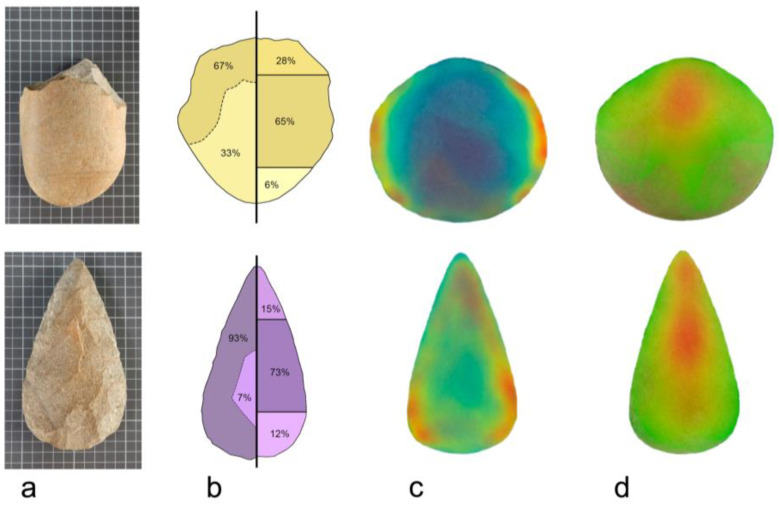
Choppers (**a**; above) and handaxes (**a**; below) trigger different visual exploration patterns. According to eye-tracking analysis, naïve subjects tend to distribute the fixations differently in the two tool types, both in terms of cortex vs. knapped surface (**b**—left drawing) and tip–center–bottom regions (**b**—right drawing). In any case, regions with high visual saliency (**c**; in red) do not capture visual attention, which is instead driven axially (**d**; red). Redrawn after Silva-Gago and Bruner (2023).

In primates (especially in anthropoids), vision is the main source of sensorial input, and hence the relationship between brain, body and environment is crucially influenced by visual attention, a complex process that involves the lateral geniculate nuclei, the pulvinar and the thalamus, the visual (occipital) cortex, and the fronto-parietal selective system (Kastner and Pinsk 2004). Visual attention is generally oriented at the functional and manipulative parts of complex tools (Pilacinski et al. 2021), a process influenced by contextual information (Federico et al. 2021) and by the familiarity with the objects (Tamaki et al. 2020). Interestingly, by using eye-tracking, we have shown that human visual attention is not driven by general visual saliency, but by functional or structural tool features, even when exploring raw stone tools like choppers or handaxes (Silva-Gago et al. 2021, 2022; Figure 3). In particular, even subjects with no experience in archeology generally direct fixations at the bottom or tip of the stone, namely, where the object is grasped (structural region for the hand-tool contact) or used (functional edge). This means that functional/structural inputs are automatically selected even where visual exploration is not associated with any specific aim, and regardless of the actual visual saliency of that region. It is reasonable to speculate that, since the Early Pleistocene (habitual tool-users), humans have undergone changes in their patterns of selective attention associated with technological integration, with possible feedbacks between selective pressure and behavioral channeling (Bruner and Iriki 2016). Namely, early specializations as habitual tool-users should have implicitly involved those attentional abilities, prompting a functional interface between brain and technological extensions (Malafouris 2010). Whether or not earlier (non-human) hominids may have used real tools, and to what extent early humans may have used smaller flakes in addition to larger choppers and handaxes, remains, at present, less clear.

The second change (obligatory tool-users) is associated with Neanderthals (*H. neanderthalensis*) and modern humans (*H. sapiens*), and it involves a pronounced reduction in general tool size and an increase in tool morphological and functional complexity (e.g., Shea 2013; Turq et al. 2013; Sharon and Oron 2014). All these changes must have been accompanied by some sort of ability to select the stimuli through a co-evolutionary process between the attentional system and the affordances of the technological elements. This is particularly true if tools are interpreted as extra-somatic peripheral components of the cognitive system, by virtue of an extended cognitive network (Kaplan 2012; Japyassú and Laland 2017). In this case, by either neuroplasticity, genetic factors, or mixed feedbacks (see Bruner and Iriki 2016), it is reasonable to think that such an ability to select the proper sensorial stimulus may be adaptive in terms of fitness. In this sense, it is worth noting that tools associated with the Lower Paleolithic period (habitual tool-users) are generally grasped with the whole hand, because of their larger size, while tools associated with the Middle and Upper Paleolithic (obligatory tool-users) are more frequently manipulated with fingers and fingertips, which suggests a profound (and finer) change of the perceptual and cognitive integration between tool and body. Therefore, on the one hand, visual attention shifts from salient regions to functional/structural regions, while, at the same time, haptic attention shifts proportionally from the whole hand to the finger, and finally to the fingertips (Bruner et al. 2018a). These changes in visual and haptic attention must have co-evolved with the changes in size (from hand-grasped to finger-grasped tools) and complexity (from scarcely knapped to profoundly knapped tools), of the technological elements. These possible changes in the orientation and filtering of the stimuli associated with tool affordances suggests that, besides *tool-using* and *tool-making*, research in cognitive archeology should be also focused on *tool-sensing* (Bruner et al. 2018b). In fact, these three aspects rely on different cognitive, behavioral and neural systems, and therefore, although they have evolved within a single and integrated technological package, they might have changed according to independent factors and with different timing. The involvement of (mainly visual and somatosensorial) perceptual factors points to *embodiment* and extended cognition as being relevant elements of our recent cognitive, cultural, and technological abilities (Wilson 2002, 2010; Borghi and Cimatti 2010; Bruner et al. 2023c). Although principles and concepts on extended or embodied cognition still lack precise theoretical definitions and a firm experimental background (e.g., Meteyard et al. 2012; Caramazza et al. 2014), it is likely that abilities and mechanisms associated with brain–body–tool integration have played a crucial role in human evolution, particularly when dealing with our own species (Bruner 2021).

### 2.2. Executive Attention

Besides potential evolutionary pressures on orienting and perceptual attention, it is nonetheless clear that major cognitive changes in the human lineages must have involved the third attentional subsystem, namely, the one in charge of executive control. In the classic model by Baddeley (2000), executive control plays a crucial role in working memory, coordinating the visuospatial and phonological buffers in goal-directed behaviors. Coolidge and Wynn (2005) proposed that enhanced working memory could have been a key factor in human cognitive evolution, and particularly in *H. sapiens*. Actually, most behavioral features uniquely expressed by modern humans within the archeological record (tool-making, hunting, harvesting and food storing, navigation and so on) do strongly rely on a consistent working memory load (Wynn and Coolidge 2010), including complex cognitive skills like those involved in aspects that are the very foundation of modern human behavior, like for example language and learning. Social cognition merits additional attention in this sense because, although in primates it depends on brain size (Dunbar 2018), it also involves a large array of inter-personal managements based on working memory constraints. Among the mechanisms sustaining most of these behaviors, a very crucial one is inhibition. In most animals, inhibition is largely based on emotional feedback (e.g., fear), while in humans it may be also associated with reasoning, motivation or cultural rules, which can disable instinctive responses without any stringent emotional load. Inhibition is deeply rooted in executive control, and it is a direct bridge to volitional and decisional networks. All these features led Wynn and Coolidge to propose that working memory (including its attentional component) may have experienced a substantial enhancement in our own species, say after 100,000 years ago. Although proficient tool-makers and competitive hunters, Neanderthals might have instead relied on long-term memory resources, which generated expert behaviors through empirical, repeated, and more automatic processes (Wynn and Coolidge 2004).

It is important to note that, according to the hypothesis of Wynn and Coolidge, modern humans did not evolve a brand new ability, but enhanced a general working memory capacity, a basic ability already present in their ancestors and in all primates. Nonetheless, there is no reason to think that the narrow and specific skills of working memory (for example, recursion; Coolidge et al. (2011)) must necessarily coevolve together, or under the same evolutionary pressure. Even if we consider only the core elements of Baddeley’s model (i.e., executive attention, visuospatial sketchpad and phonological loop), we can imagine that different species can evolve different combinations of these three cognitive resources. Therefore, when considering working memory and human evolution, we must take into account the possibility that distinct hominid species might have differed not only in terms of magnitude (more or less working memory capacity), but also in terms of cognitive structure (different weights and contributions of the working memory components). This possibility must necessarily change the perspective with which we interpret the behavior associated with the archeological record.

Two additional aspects must be further considered when integrating cognitive issues related to executive attention with evolutionary diversity. First, executive functions play a key role in working memory, but they are involved in many processes that go far beyond the short-range management of visual and phonological information. For example, when dealing with behavioral flexibility and inhibition, executive functions are entrenched with a large set of autobiographical (long-range) factors that rely on more distributed cognitive abilities (see below). Second, an optimal behavior to optimize resource exploration and exploitation requires a proper balance between flexibility and inhibition, and between automatic (unaware) and deliberated (aware) responses. Interestingly, such balance is influenced by a fine regulation of tonic and phasic arousal, which in turn depends on an interplay between the alerting and the executive system (Rueda et al. 2023). This inevitably brings forth several possible combinations of sensitivity to top-down and bottom-up effects, according to the traditional view of attention as a double-flow process (Baluch and Itti 2011; Chica et al. 2013; Krauzlis et al. 2021).

### 2.3. A General View

In sum, we can speculate that the evolution of the genus *Homo* (and, in particular, of our own species, *H. sapiens*) has been characterized by important changes in the attention system. If we rely on broad indicators hypothetically associated with increased attentional capacity, we can at least provide the following general list of features that may be correlates of attentional changes (see also Bruner and Colom 2022):

1. The relative brain size (encephalization) did increase with the origin of the human genus (*H. ergaster* and *H. erectus*), then again during the Middle Pleistocene (*H. heidelbergensis*), and finally in modern humans (*H. sapiens*) and Neanderthals (*H. neanderthalensis*). However, it is important to take into account that this increase deals with average values, while the range of brain size in all these species does largely overlap. Furthermore, considering that correlations between brain size and most cognitive features range from null to modest, mean species differences are not really informative on possible individual differences;
2. Technological complexity is similar in *H. erectus* and *H. heildelbergensis*. Then, it displays a general increase in *H. neanderthalensis* and early *H. sapiens*, and a surprising boost in late modern humans (say in the last 50,000 years). Technological complexity is supposedly linked to distinct cognitive aspects, including reasoning, memory, and attentional skills;
3. Similarly, social complexity (group size, social structure, landscape use, economy, hunting strategy, etc.) underwent an apparent increase in *H. neanderthalensis* and early *H. sapiens*, and then a pronounced escalation in the latter lineage.

These three aspects are undoubtedly associated with a large array of cognitive abilities, and we must hence assume that in all these cases, attention must have undergone profound enhancements. All these changes are intricately embedded in the social and technological aspects of our peculiar evolution, and must have concerned both perceptual and executive abilities (Bruner and Gleeson 2019; Osiurak et al. 2022). In this sense, as mentioned, attentional changes have likely involved both bottom-up and top-down mechanisms. Although these two distinct modes of attentional selection can be distinguished at both the functional and phylogenetic level (Klein and Lawrence 2011; Posner 2023), their respective contributions can be harder to disentangle in evolutionary terms, when taking into account their interactions and reciprocal influences (Stout 2010).

Besides the many details still to be investigated in this general scenario, it remains to be evaluated what biological components have been selected according to the evolving cognitive requirements. We have to remember that selection works strictly on reproductive success, the number of offspring being the unique parameter employed to consider whether a package of traits is advantageous or detrimental. Because of the many elements involved, the answer is likely to be complex and complicated. Direct genetic influences cannot be ruled out when dealing with perceptual abilities and executive skills, or more generally with the integration capacity of the association cortex (Buckner and Krienen 2013). Nonetheless, because of the complex interactions between biological and cultural evolution, more complex scenarios can be also considered, in which genes, brain, bodies and tools coevolved through feedbacks based on neuroplasticity and environmental effects (like the Baldwin effect; see Sznajder et al. 2012; Corbey 2020). Neuroplasticity is indeed a key factor in human brain evolution (Sherwood and Gómez-Robles 2017), and this might be particularly relevant when considering the sensitivity of attention to training and environmental conditions, especially when dealing with executive control (Tang and Posner 2009).

## 3. Fronto-Parietal Anatomy and the Human Genus

It is not clear whether changes of the attentional system through the hominid lineages can leave apparent traces on the macroscopic cortical phenotype, as can be observed from endocranial casts. Despite repeated (and scarcely documented) claims in dissemination venues, the frontal lobes, which are a crucial region involved in executive control, have not revealed any substantial morphological differences between human species (Bruner and Holloway 2010). Modern humans and Neanderthals have somewhat proportionally wider frontal lobes than more archaic hominids, but this could easily be the secondary result of spatial constraints exerted by the overlap and tight contact of the prefrontal cortex with eyes and orbits in these two species, which limits the vertical growth of these cortical regions (Bruner et al. 2014). Specific frontal cortical features that can be derived in the genus *Homo* are debated, because they are associated with faint sulcal traces inferred from the endocranial surface, with traits that display a noticeable (and scarcely known) individual variability, and with cranial (bone) references that indeed are not informative of the extension and proportions of brain cortical regions (e.g., Ponce de León et al. 2021). When compared with other primates, humans definitely display some specialization in localized prefrontal regions, and probably more connections (Sherwood and Smaers 2013). However, such details cannot be recognized in fossil specimens, mostly if we consider that the gross frontal proportions are the same for humans and apes (Semendeferi et al. 2002). In sum, fossils are not able, at present, to reveal any patent macroscopic change of the frontal lobes along the human lineages. Taking into account the importance of the prefrontal cortex in executive attention, this must be interpreted as an absence of evidence, and not evidence of absence.

Furthermore, deep cerebral regions that are central to the attention system (like the cingulate cortex) cannot obviously be investigated in extinct species, and any speculation in this sense is hence indirect. Extrapolations on morphological changes of these deep brain regions can be tentatively inferred because of external changes. For example, the cingulate gyrus is an important topological bridge between the anterior and posterior cerebral districts and, accordingly, external spatial rearrangements must be necessarily associated with consensual internal modification (Bruner 2022b). Nonetheless, such an approach is largely theoretical and speculative, and difficult (or impossible) to test on fossils.

Therefore, there is no agreement on whether or not we can detect in fossil humans patent morphological changes of the frontal regions of the brain. The lack of clear evidence, despite the many efforts devoted to this topic, suggests that, if humans underwent some gross anatomical changes of the anterior cortex, these must have been subtle, or at least silent to the fossil record.

In contrast, the parietal cortex (a region that is crucially involved in the attentional network) has displayed evident morphological variations through the human phylogeny. The parietal cortex is particularly expanded, diversified and connected in primates, and particularly in *H. sapiens* (Goldring and Krubitzer 2017). The parietal lobe is a major cerebral hub in terms of functional and structural networks (Hagmann et al. 2008), and it is characterized by an intense metabolic load (Vaishnavi et al. 2010). In general, if we compare the morphological variation among primates and within the human genus, the parietal lobes are wider in Neanderthals, and much more expanded in modern humans (Bruner 2018). Part of such shape variation might be due to geometrical and structural changes in the cranial architecture (Zollikofer et al. 2022). However, when cerebral (lobe) anatomical references are used instead of cranial (bone) metrics, there is also evidence of a real expansion of the external cortical surface (Bruner 2004; Pereira-Pedro et al. 2020). In *H. sapiens* and *H. neanderthalensis*, the inferior parietal lobule and the intraparietal sulcus have likely been involved in these macroscopic changes. It is worth noting that, among the many functions these areas are involved in (including eye–hand coordination), they also look crucial for technical reasoning (Federico et al. 2022). However, in the case of modern humans, we can additionally speculate a further contribution of the precuneus, which is also much expanded in *H. sapiens* when compared with apes (Bruner et al. 2017a; Willbrand et al. 2023). The precuneus, in terms of architecture, includes the superior parietal lobule, and it is generally separated into an anterior, a middle, and a posterior area (Scheperjans et al. 2008). The anterior region is more involved in somatic integration, the posterior one in visual integration, and the middle part is a bridge between body perception and vision (Cavanna and Trimble 2006; Margulies et al. 2009; Zhang and Li 2012). Such parcellation is in agreement with cortical gradients (Huntenburg et al. 2017)—in this case a pretty linear gradient—between somatic and visual perceptual regions. In this sense, the precuneus can be interpreted as a peculiar association region, because of its intimate connection with and dependence on sensorial inputs (in contrast with the non-canonical organization of many association areas; Buckner and Krienen 2013). Interestingly, general spatial models suggest that the diversity and variation of the precuneus among adult modern humans might be associated with its anterior/somatic regions (Bruner et al. 2017b), while phylogenetic differences (that is, with Neanderthals and apes) could be more localized in its posterior/visual portion (Bruner et al. 2017a; Pereira-Pedro et al. 2020). Although a linear or simple association between cortical morphology and cognitive functions is not certain, we should consider the possibility that such cortical expansion may actually be associated with some kind of cognitive and behavioral aspect. Those areas are involved in visuospatial integration, and, by chance or not, these morphological changes match, in modern humans, the evolution of species-specific visuospatial behaviors, like graphic, manipulative, and throwing abilities (Bruner et al. 2018c). Indeed, the integration between somatic and visual perception is also the very foundation of the self, taking into account that the body is the main unit for any mental experiment or representation relying on physical, chronological, social, or mnemonic spaces (Hills et al. 2015; Land 2014; Faivre et al. 2015; Maister et al. 2015; Peer et al. 2015).

## 4. The Parietal Lobe, Attention, and the History of the Self

The parietal lobe is a crucial element of the attentional system, as part of a specialized fronto-parietal network (e.g., Behrmann et al. 2004; Raz 2004; Bisley and Goldberg 2010; Petersen and Posner 2012; Scolari et al. 2015). Neuropsychological evidence, in particular, has long highlighted a relationship between the parietal cortex and spatial attention (Posner et al. 1984; Corbetta et al. 2005). The parietal cortex is central to the attention/intention system, which, although partially conserved through primates (Rushworth et al. 2001), displays specialized connections in humans, concerning both the inferior and superior lobules (Hecht et al. 2013; Ardesch et al. 2019). It is therefore reasonable to think that there could be an association between the neuroanatomical changes of the parietal cortex in *H. sapiens* and its attentional capacity, most of all when dealing with issues related to visual, somatic, and spatial attention. According to the information from the paleontological and archeological records, we probably cannot speculate too much about what features, areas or abilities have been precisely involved in such changes, and the enhancement or specialization of the parietal functions may have spanned from selective filtering to attentional awareness, which are fundamental aspects of inhibition and volition. These same functions are also crucial for the evolution of proper self-perception, involving ecological (physical environment), interpersonal (social), extended (chronological), private (feelings, thoughts and intentions) and conceptual (abstract and symbolic representation) aspects of consciousness (Leary and Buttermore 2003).

However, the parietal lobes (and, in particular, the precuneus, the supramarginal gyrus and the angular gyrus) are also implicated in a distinct system: the default mode network (Buckner and DiNicola 2019). This network is generally associated with introspective and off-line thinking, past and future projections, and mind-wandering. Self-centred thinking, mental simulations and internal models, necessarily rely on visual (imaging) or speech (language) components, which have some patent overlap of resources with, respectively, the visuospatial and phonological elements of the working memory system. Differently from working memory, however, mind-wandering is completely off-line. In other words, it is disconnected from the sensorial feedback, and not coupled with real (i.e., physical, present) environmental events. Such a capacity for off-line thinking is constrained by the capacity of past and future projections, which constitutes the very foundation of the ego, through the generation of a chronological narrative based on (visual and linguistic) memories and predictions. The extension and complexity of such mental projections determine the extension and complexity of such narrative, and, therefore, of the self.

**Figure 4:**
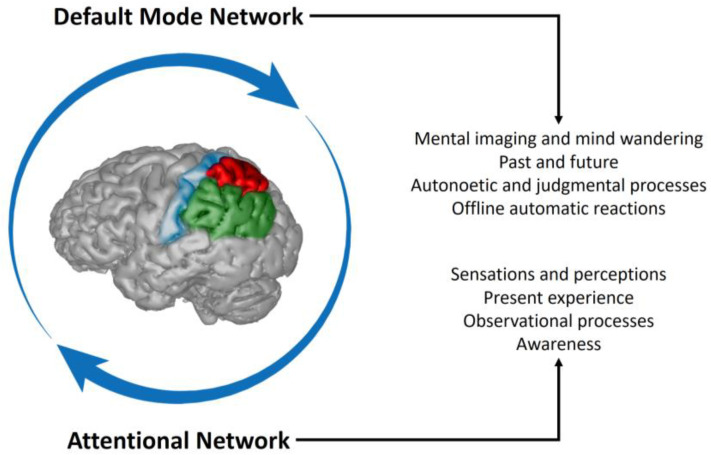
The Default Mode Network mainly deals with off-line egocentric narratives based on mental imaging and internal speech, while an important part of the attentional network concerns on-line perception and present moment awareness. The parietal lobes have a key role in both processes, and a proper cognitive efficiency depends on an accurate balance between these two systems. Meditation practice improves the ability to recognize mind-wandering and return to a present-moment attentional state.

This skill is of course an outstanding ability of modern humans and, as any specialization, may involve some drawbacks. Mind-wandering, as a task-unrelated or stimulus-independent mental activity, is associated with distinct psychological processes, including creativity or dreaming (Christoff et al. 2016). However, it has a straightforward adaptive value when employed to maximize survival through risk reduction, and this is probably the evolutionary basis of the well-known “negativity bias”, which is particularly stressed in young ages (Carstensen and DeLiema 2018). Put simply, this cognitive bias deals with the fact that negative aspects capture more attention than the positive ones. In terms of evolution, this can be adaptive because it prevents risks and dangers. Nonetheless, in terms of individual psychological stability, it can be overemployed and lead to pathological and sub-pathological conditions. An excess of mind-wandering is in fact associated with anxiety, depression, or, generally, with ruminations that can sensibly decrease the quality of life (Killingsworth and Gilbert 2010; Chaieb et al. 2022). That is, past and future projections do habitually generate fears, sadness, anguish, uncertainties, resentment, and a large array of negative emotions that trigger distress and suffering, a common world-wide human condition recognized by most philosophical traditions. The ubiquity of this unbalance may suggest that it can represent a human universal, which, following the principles of human ethology, are intrinsic features of a species’ biology and behavior rooted within its phylogenetic and adaptive history (Eibl-Eibesfeldt 1989). If so, we can wonder whether the evolutionary development of the parietal cortex in modern humans may have implied a sort of mismatch between the attentional and the default mode systems. Such an imbalance can lead to an excess of rumination and mind-wandering, or, put differently, to an attentional ability that, although specialized and enhanced, is not able to regulate properly the (even more outstanding) visuospatial and linguistic capacities (Figure 4).

In this sense, it is worth mentioning meditation, a practice (or an assortment of diverse practices) that is often aimed at training the mind to escape off-line egocentric projections and return to the perceptual present experience. Meditation relies on an extended fronto-parietal network (Tang et al. 2015), through the activation of different kinds of attentional abilities (Lutz et al. 2008; Dahl et al. 2015; Sumantry and Stewart 2021). Many meditation practices, indeed, are based on a cyclic shift between attentional and default mode networks, in which the training aspects deal with the recognition of a wandering state of mind and a successive intentional shift to the attentional system (Brewer et al. 2011; Hasenkamp and Barsalou 2012; Hasenkamp et al. 2012).

**Figure 5:**
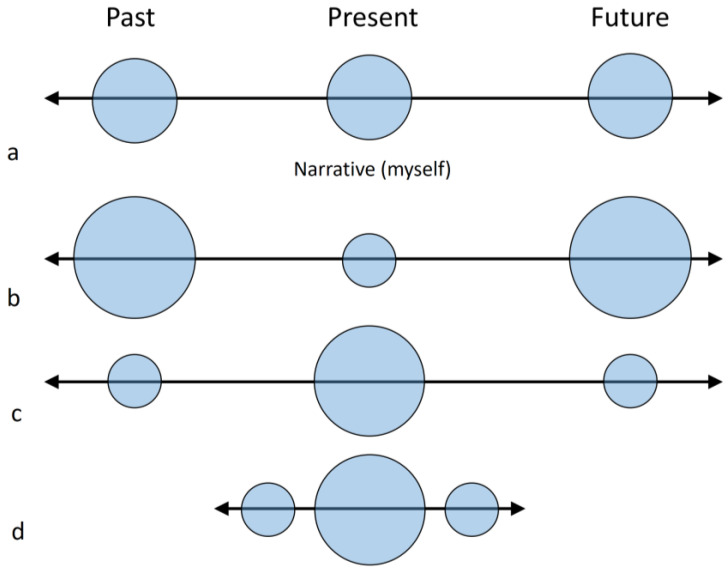
Self-narrative is based on the capacity to extend past and future projections through mental imaging, speech and memories (**a**). An excess of ruminations may generate an over-representation of past and future projections, disconnection from the present moment and disproportionate mind-wandering (**b**). Meditation is a form of cognitive training aimed at focusing on the experience of the present moment, through proper control of the attentional resources (**c**). We might speculate that limitations in visual imaging and language (as hypothesized for extinct human species) could be associated with shorter and simpler self-narrative, which reduces mind-wandering but, at the same time, hampers a sustained and intentional capacity to support attentional awareness (**d**). These different conditions are likely to influence most aspects of the psychological, ecological, technological and social life of a person/species.

As mentioned above, both the archeological and paleontological records suggest that extinct human species may have lacked the modern human capacity for working memory and visuospatial integration. The smaller parietal cortex, the scarce or null graphic and throwing abilities, the reduced group size and much other evidence associated with visual, linguistic or attentional behaviours, are at least compatible with the hypothesis of a less developed fronto-parietal complexity in non-modern human species, when compared with *H. sapiens*. In this sense, we can therefore speculate whether these taxa may have relied on “shorter and simpler” egocentric narrative (Figure 5), depending more on (on-line) perceptual and emotional feedback and less on (off-line) memories and expectations.

When trying to delineate the cognitive range of extinct minds, it is important to consider that, although there might be uncertainties regarding the precise relationship or overlap between attention and awareness, the former is probably necessary to the latter (see Lamme 2003; Robertson 2003; Cohen et al. 2012). In particular, we can expect that a *sustained* and *intentional* awareness must necessarily rely on attentional, executive and volitional processes. The term “sustained” refers to a prolonged cognitive activity, and the term “intentional” concerns willingness and disposition. Attentional capacity, in this case, is at least indispensable to warrant a proper filtering of bottom-up stimuli and the orientation of the top-down focus. The reasons behind such conscious attentional involvement are of course rooted in a proper (enduring) narrative of the self, based on visual, linguistic, and mnemonic resources. As mentioned, the precuneus is central to coordinate vision and the body into a congruent self (Faivre et al. 2015), and to integrate this self-representation into the wider frame of episodic memory (Lou et al. 2004; Northoff and Bermpohl 2004; Land 2014). In this sense, therefore, we can speculate that the on-line (present moment) experience in extinct human species, despite preventing invasive mind-wandering, may have lacked a modern-like state of consciousness, namely, a level of self-narrative and intentional awareness comparable, in terms of both extension and complexity, with *H. sapiens*.

## 5. Limitations and Concluding Remarks

Working with cognition and behavior in extinct species is implicitly a speculative and incomplete task. Most hypotheses presented in this perspective review can be tested only indirectly, by using modern humans as models in experimental psychology, functional neuroimaging, and comparative neuroanatomy. Apart from a pure theoretical approach, all we can do is use current evidence in neuroscience and psychology to delineate reasonable perspectives, namely, scenarios that are compatible with the available observations and data. This is, after all, the general rule in science, and in this sense working with extinct species is not much different than working with extant ones. Nonetheless, when dealing with human evolution, we should probably employ more conservative approaches, so as to avoid a counterproductive excess of speculations. Because of the implicit conflict of interests when dealing with our own natural history, we should also take into account some additional cautions. On the one hand, we should try to avoid a traditional anthropocentric and deformed view of phylogeny, which presents evolution as a linear, gradual and progressive trend, moving from more inefficient to more proficient species and culminating in our own. In this sense, it is once more worth noting that all extinct species had their own derived features, namely, cognitive or behavioral traits that we may have lost or never evolved. At the same time, we should avoid falling into the tendency to deny differences and their evidence, in order to circumvent alleged politically incorrect specisms. For example, Neanderthals and modern humans, despite having evolved for a similar amount of time and showing the same brain size, have displayed outstanding differences in their respective cultural and technological abilities, which suggests a noticeable increase in cognitive complexity in our own lineage (Wynn et al. 2016). We must admit that the archeological record is biased by a chronological factor: the older the age, the fewer remains we find. Nonetheless, in the case of modern humans and Neanderthals, this limitation is less stringent, because the two lineages were roughly concurrent. Differences, variations and variability are the very foundation of evolution, and rejecting differences means refusing the possibility to understand the evolutionary process. The recognition of such differences might be, indeed, an easy task. Of course, difficulties come when trying to understand when, why and how such differences emerged. The problem deals with the fact that the paleontological (fossils) and archeological (culture) records are not only dramatically incomplete, but also scarce, which generally hampers any consistent quantitative analysis of the features involved. Nonetheless, difficulties with these topics are also due to the implicit complexity of the cognitive process itself. For many aspects associated with attention, we still lack substantial information on factors, variables and parameters in our own species. Concepts and definitions are, at best, vague, tentative or too comprehensive, generating disagreements and paradoxes when applied to specific circumstances. Attention itself is a very broad term, which includes very distinct processes, abilities, and neuronal circuits. On the one hand, attention can be generally interpreted as a limiting factor for all the other cognitive skills. At the same time, it deals with the delicate scope of selecting information for a system that has inherent processing constraints. Both these wide and narrow functions are, indeed, crucial for survival and reproduction, and deeply influence the ecological and cognitive niche. With all these limitations in mind, an evolutionary perspective can supply inspiring hypotheses that, although provisional and imprecise, can provide a different (and fruitful) view of our current cognitive landscape.
